
JOURNAL INFORMATION
==============================
NLM Title Abbreviation: J High Energy Phys
Journal ID: J High Energy Phys
NLM Journal ID: 101668727

Journal of High Energy Physics

ISSN: 1126-6708
EISSN: 1029-8479

ARTICLE INFORMATION
==============================
PMCID: PMC8827340
PMID: 35226710
DOI: 10.1007/JHEP05(2015)012
Article ID: 1232
Article version: 1
Subjects: Erratum

Erratum to: AdS6 solutions of type II supergravity

Apruzzi Fabio fabio.apruzzi@itp.uni-hannover.de
1
Fazzi Marco mfazzi@ulb.ac.be
2 3
Passias Achilleas achilleas.passias@unimib.it
4 5
Rosa Dario dario.rosa@mib.infn.it
4 5
Tomasiello Alessandro alessandro.tomasiello@unimib.it
4 5
1 0000 0001 2163 2777 grid.9122.8 Institut für Theoretische Physik, Leibniz Universität Hannover, Appelstraße 2, 30167 Hannover, Germany
2 0000 0001 2348 0746 grid.4989.c Physique Théorique et Mathématique, Université Libre de Bruxelles, Campus Plaine C.P. 231, B-1050 Bruxelles, Belgium
3 0000 0001 2189 8962 grid.425224.7 International Solvay Institutes, Campus Plaine C.P. 231, B-1050 Bruxelles, Belgium
4 0000 0001 2174 1754 grid.7563.7 Dipartimento di Fisica, Università di Milano-Bicocca, Piazza della Scienza 3, I-20126 Milano, Italy
5 grid.470207.6 0000 0004 8390 4143 INFN, sezione di Milano-Bicocca, Piazza della Scienza 3, I-20126 Milano, Italy
Electronic publication date: 2015 May 4
Print publication date: 2015
Volume: 2015
Issue: 5
First page: 1
Last page: 1
Received 2015 Apr 3; Accepted 2015 Apr 3
Copyright: © The Author(s) 2015
License: Open AccessThis article is distributed under the terms of the Creative Commons Attribution 4.0 International License (https://creativecommons.org/licenses/by/4.0), which permits use, duplication, adaptation, distribution, and reproduction in any medium or format, as long as you give appropriate credit to the original author(s) and the source, provide a link to the Creative Commons license, and indicate if changes were made.
License URL: https://creativecommons.org/licenses/by/4.0/

Erratum for: 10.1007/JHEP11(2014)099
issue-copyright-statement: © SISSA, Trieste, Italy 2015

==============================
Open Access

This article is distributed under the terms of the Creative Commons Attribution License (CC-BY 4.0), which permits any use, distribution and reproduction in any medium, provided the original author(s) and source are credited.

Erratum to: JHEP11(2014)099

ArXiv ePrint: 1406.0852

